# From concept to regulation: OECD’s role in the development of the DNT *in vitro* battery

**DOI:** 10.3389/ftox.2026.1774469

**Published:** 2026-03-20

**Authors:** Magdalini Sachana, Helena T. Hogberg, Iris Mangas

**Affiliations:** 1 Environment Health and Safety Division, Environment Directorate, Organisation for Economic Cooperation and Development (OECD), Paris, France; 2 National Toxicology Program Interagency Center for the Evaluation of Alternative Toxicological Methods, Division of Translational Toxicology, National Institute of Environmental Health Sciences, Research Triangle Park, NC, United States; 3 Pesticides Peer Review Unit, European Food Safety Authority, Parma, Italy

**Keywords:** defined approaches, developmental neurotoxicity, *in vitro* battery, integrated approaches to testing and assessment, NAMs, regulatory application

## Abstract

Chemical exposure during critical stages of nervous system development can lead to adverse neurotoxic effects, known as developmental neurotoxicity (DNT). Although standardized *in vivo* test guidelines (TGs) are required under various regulatory frameworks, compared to other TGs few *in vivo* DNT studies have been conducted. The complexity, interpretative limitations, high cost and resource demands of these studies have prompted regulatory agencies and stakeholders to emphasize the need for developing and standardizing NAMs (New Approach Methodologies) that capture relevant endpoints and biomarkers of DNT effects. Recent advances in *in vitro* neural models and high throughput analytical technologies have enabled the creation of a battery of assays -the DNT *In Vitro* Battery (DNT-IVB)- designed to rapidly assess chemicals for their impact on critical neurodevelopmental processes in *in vitro* test systems. In response to the regulatory needs and the scientific advancements, an international collaboration led by regulatory agencies from both sides of the Atlantic under the auspices of OECD was initiated about a decade ago. Since then, substantial progress has been made. The DNT-IVB provides a structured approach for integrating human-relevant *in vitro* assay data into chemical DNT assessments. Its application is supported by guidance in the OECD Initial Recommendations for evaluating DNT-IVB data, along with case examples that apply the IATA (Integrated Approaches to Testing and Assessment) framework. Ongoing efforts aim to address remaining challenges and consolidate the guidance needed for standardizing and expanding the application of NAMs to assess DNT across various regulatory contexts. The DNT-IVB now represents a flagship OECD project, testing the paradigm of applying NAMs and IATA in risk assessment and paving the way for broader worldwide alignment on NAMs standardization and use.

## 1 Introduction

Neurodevelopmental disorders (NDDs) include a wide range of conditions that involve intellectual disabilities, communication disorders, Autism Spectrum Disorder (ASD), Attention-Deficit/Hyperactivity Disorder (ADHD), specific learning disorders, and neurodevelopmental motor and sensory disorders (APA, 2022). While the etiology of these NDDs is unclear, it is suggested that the origin may be due to a combination of environmental and genetic factors (De Felice et al., 2015; Griffin et al., 2022; Khodosevich and Sellgren, 2023). These disabilities can adversely affect academic and occupational performance at an individual level. At population level, NDDs can have negative public health and socioeconomic consequences, which are of deep concern at both societal and political levels. In the last decades, there has been a significant increase in these disorders (Hogberg et al., 2025; Li et al., 2023). This rise could partly be due to broadening of diagnoses and access to healthcare; however, this is likely not the sole cause of increased incidence of NDDs (US EPA, 2024a). Neurotoxicity is a perturbation to the function, structure, or the chemistry of the nervous system, while developmental neurotoxicity (DNT) includes any of these types of changes that occur following exposure during nervous system development. Neurotoxicity can include a wide range of pathological changes leading to behavioral changes, myelinopathy, gliopathy, neurodegeneration, axonopathy, and various forms of neurochemical and/or neurophysiological dysfunction or disruption (OECD, 2007; US EPA, 1996). There is concern that certain chemicals may increase their prevalence because of the unique susceptibility of the developing nervous system, which can be especially sensitive to these substances (Grandjean and Landrigan, 2006; 2014; Luo et al., 2025; Zaks et al., 2025; Cohen-Eliraz et al., 2025; Zhang et al., 2025; Su et al., 2025). The developing nervous system is more sensitive than the adult nervous system because neurodevelopment is a complex and finely controlled process, and its disruption can result in long-term changes in neuroanatomy and brain function (Giordano and Costa, 2012; Heyer and Meredith, 2017; Rodier, 1995). Although they cannot provide causative linkages, epidemiological studies have shown associations between exposure to specific chemicals and the rising rates of NDDs (De Felice et al., 2015; Bölte et al., 2019). The increase in incidence of NDDs has increased public awareness and concern about DNT following chemical exposure. Thus, this endpoint is of both regulatory and public concern.

DNT is an essential endpoint in chemical safety assessment and is embedded in multiple national and regional regulatory frameworks (Boyd et al., 2025; Viviani et al., 2025). Recognizing its vulnerability and safeguarding the developing brain from potentially hazardous chemical exposures led to the development of *in vivo* test methods for evaluating chemical exposure effects on brain development using rodent animal models in the 1980s. These initial efforts led to an *in vivo* Test Guideline (TG) published by the US EPA in 1991, updated in 1996 (US EPA, 1991; US EPA, 1996), which was refined as an international OECD TG in 2007 (OECD, 2007). It is designed for screening potential chemical-induced DNT hazards, measuring both behavioral (i.e., clinical signs, ontogeny of motor function, startle response, and learning and memory) and neuropathological endpoints (including neurophysiological changes and morphometric measurements). However, the use of the US EPA (US EPA, 1996), NAFTA (NAFTA, 2016) and OECD DNT *in vivo* TGs (OECD, 2007; OECD, 2018a) have been limited compared to other guideline toxicity studies, potentially due to their methodological complexity, biological differences in neurodevelopment between humans and rodent animal models, ethical concerns and high costs (Crofton and Mundy, 2024; Mangas et al., 2025a).

Developing and identifying appropriate animal models for DNT testing has proven challenging. First, the nervous system exhibits greater degree of cellular, structural, and chemical heterogeneity (e.g., neurotransmitters) compared to other organ systems. Second, toxic chemicals can affect any functional or structural component of the nervous system, leading to adverse outcomes such as sensory and motor deficits, memory impairment, behavioural changes, and neurological abnormalities. These effects vary across species and are strongly influenced by the developmental window of exposure. Third, unlike rodents, the human nervous system undergoes a prolonged developmental period that extends beyond birth, rendering it more vulnerable to environmental chemical exposures during fetal and early postnatal stages. Finally, the relatively long human lifespan increases the likelihood of cumulative exposures, while additional modulatory factors complicate the final response. As a result, several neuronal functions cannot be meaningfully evaluated using rodent models (Serafini et al., 2024).

At the same time, new NAMs have emerged as animal-alternative strategies to advance chemical hazard and risk assessment (US EPA, 2016; US EPA, 2021). To provide improved human predictivity of hazard identification and characterization, the shift to approaches and methods that utilized human relevant models was initialized in the field of toxicology. Building on the Toxicity Testing in the 21st Century report, NAMs integrate *in vitro* systems, *in silico* modeling, high-content screening, and non-mammalian assays to address the limitations of traditional *in vivo* studies by incorporating human-relevant models into medium- to high-throughput and computational approaches (Richard et al., 2016; Judson et al., 2014; European Union, 2009; Escher et al., 2022; European Commission, 2023; Truong et al., 2025; Paul Friedman et al., 2025). As the science underlying regulatory decisions shifts away from traditional *in vivo* models and towards implementing NAMs and related *in vitro* approaches, it is imperative that confidence in these approaches be established to ensure regulatory adoption (OECD, 2005).

The OECD has published documents on *in vitro* test methods and has provided additional support for NAMs through several guidance documents and international coordination on numerous efforts. For example, OECD 211 “Guidance Document for Describing Non-Guideline *In vitro* Test Methods” in 2017 (OECD, 2017a). Similarly, the OECD published a “Guidance Document on Good *In Vitro* Methods Practices (GIVIMP) in 2018, further supporting *in vitro* approaches (OECD, 2018b). These guidance documents offer a flexible and comprehensive strategy to aid in the communication between developers of *in vitro* NAMs and those involved in regulatory decision-making who use NAM data (OECD, 2017a; OECD, 2018b). This paradigm shift is especially critical for DNT, where existing guideline studies are costly, resource-intensive, and difficult to interpret, resulting in the vast majority of chemicals remaining unevaluated.

Challenges in performing and interpreting *in vivo* DNT studies for predicting human developmental neurotoxicity prompted a series of collaborative workshops between research and regulatory communities initiated in 2005 by the European Centre for the Validation of Alternative Methods (ECVAM) and jointly organized by the European Chemical Industry Council, and the Johns Hopkins University Center for Alternatives to Animal Testing (see Table 1 in Sachana et al., 2019).

**TABLE 1:** KNPs measured, assays and test systems in the DNT-IVB (OECD, 2023). See OECD (2023) for references and ToxTemps details of the assays.

Key neurodevelopmental process	Assay	Test system	Assay duration chem exposure	DNT endpoint
Proliferation	NPC1	Human NPC grown as proliferating 3D neurospheres	72 h/72 h	Neurosphere area, BrdU incorporation in dividing cells
	hNP1 Prolif	Human NPC	24 h/24 h	BrdU incorporation in dividing cells
Apoptosis	hNP1 Apop	Human NPC	24 h/24 h	Apoptosis pathway (Caspase) activation
Migration	UKN2	Human NSC-derived neural crest cells	72 h/24 h	Number of cells moving into defined area
	NPC2a	Human NPC grown as differentiated 3D neurospheres	72 h/72 h 120 h/120 h	Mean distance of radial glia (nuclei negative for neuronal and oligodendrocyte markers) from edge of sphere
	NPC2b	Human NPC grown as differentiated 3D neurospheres	120 h/120 h	Mean distance of tubulin-positive neurons from edge of sphere
	NPC2c	Human NPC grown as differentiated 3D neurospheres	120 h/120 h	Mean distance of O4-positive oligodendrocytes from edge of sphere
Neuronal differentiation	NPC3	Human NPC grown as differentiated 3D neurospheres	120 h/120 h	Number of tubulin-positive neurons
Neurite outgrowth	NPC4	Human NPC grown as differentiated 3D neurospheres	120 h/120 h	Neurite length and area
	UKN4	Human NSC-line (v-myc transformed)	72 h/24 h	Neurite area
	UKN5	Human iPSC-derived peripheral (sensory) neurons	24 h/24 h	Neurite area
	hN initiation	Human NPC-derived neurons	48 h/48 h	Neurite length
	Cortical initiation	Rat primary neocortex	48 h/48 h	Neurite length
Neurite maturation and synaptogenesis	Cortical maturation	Rat primary neocortex	288 h/120 h	Dendrite length
	Cortical synapto	Rat primary neocortex	288 h/120 h	Synapse number
Glial differentiation	NPC5	Human NPC grown as differentiated 3D neurospheres	120 h/120 h	Number of O4-positive oligodendrocytes
Neural network formation	Cortical MEA	Rat primary neocortex	288 h/288 h	Action potential spike and burst parameters related to network connectivity

During these years, considerable scientific and regulatory advancements in developing and standardizing DNT assessment have been achieved. In the early Workshops some general agreements emerged: 1. due to the complexity of the nervous system development efforts focused on the development of phenotypic screens that evaluates key neurodevelopmental processes (Lein et al., 2007); 2. a single assay would not sufficiently cover the biological complexity of neurodevelopment and a battery of assays was proposed (Lein et al., 2005; 2007) 3. the assumption was that chemical perturbation of these key processes may result in adverse DNT outcomes. The objectives of these workshops were first to identify fundamental neurodevelopmental processes of the human brain and develop human-relevant DNT *in vitro* assays to model and measure these processes. These *in vitro* assays were subsequently assembled into a battery that, once used to generate data, could be combined by applying the integrated approaches to testing and assessment (IATAs) framework (OECD, 2023; OECD, 2022a; OECD, 2022b; OECD, 2022c; OECD, 2022d; OECD, 2022e).

The early scientific Workshops and the regulatory necessity led to a movement to implement NAMs into chemical DNT assessment, by EFSA, and US EPA, overseen by OECD (OECD, 2017b) with the establishment of an OECD DNT-IVB expert group in 2018 (Sachana et al., 2021).

This paper provides an overview of the historical development of NAM based DNT testing, with a focus on the achievements, confidence-building, lessons learned, and remaining challenges addressed during almost a decade of work by the OECD DNT-IVB Expert Group. It also highlights ongoing efforts in DNT *in vitro* testing and describes how OECD projects aim to deliver tools that support regulatory uptake and contribute to the establishment of DNT Integrated Approaches to Testing and Assessment (IATA) Standard Frameworks and Defined Approaches (DAs)^1^. Further information on progress within the OECD Chemicals Program, which is advancing standardized, human relevant, alternative approaches to assess chemicals that may affect the developing nervous system, is available on the OECD webpage dedicated to DNT activities (https://www.oecd.org/en/topics/sub-issues/testing-of-chemicals/in-vitro-assays-for-developmental-neurotoxicity.html).

## 2 Establishing a NAM-based DNT testing approach

**FIGURE 1:**
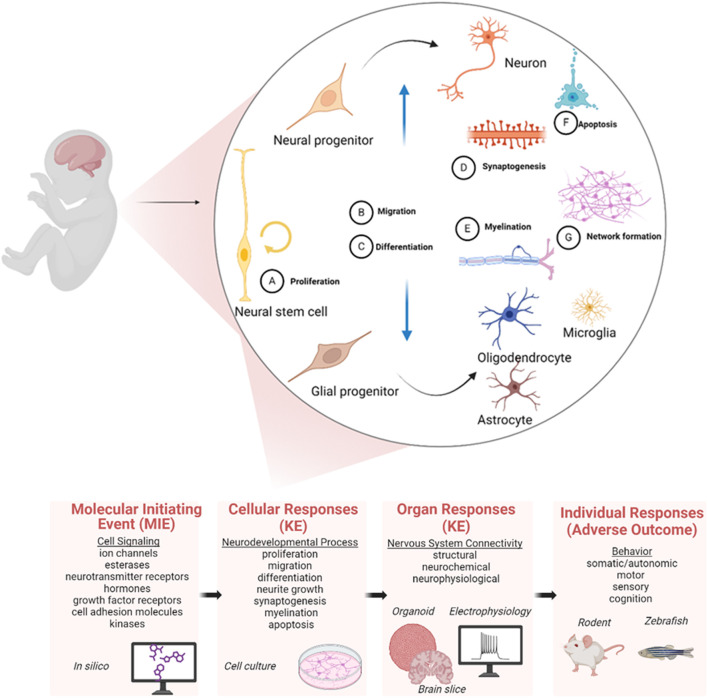
Key neurodevelopmental processes necessary for nervous system development captured in the DNT-IVB as represented within a conceptual AOP. The current DNT-IVB measures many of these processes (adapted Figure 2 of OECD, 2023), which may be altered by exposure to chemicals. Changes in any of these key processes can alter the brain structure and function of the developing organism, leading to behavioral changes. The bottom panel depicts the position of these neurodevelopmental processes as cellular key events (KEs) within the context of the Adverse Outcome Pathway framework [adapted from OECD, 2023].

The limited *in vivo* DNT testing, the recent advances using *in vitro* neural models combined with technologies for higher throughput analysis and the global movement away from using animals for testing chemical safety have fostered calls for the development of NAMs focused on rapidly assessing chemicals for potential DNT. Finding suitable non-animal methods based on *in vitro*, *in silico*, or alternative species (e.g., zebrafish, *C. elegans*, planaria, etc.) requires significant effort, even for less complex endpoints than DNT (Casati et al., 2022; Daniel et al., 2025). Neurodevelopment involves three general stages of cell proliferation, migration, and differentiation. As the CNS develops, diverse subpopulations of brain regions progress at different rates during which processes like neurogenesis, synaptogenesis, neurotransmission, glial cell maturation/development, and synaptic pruning are vital to development (i.e., Key Neurodevelopmental Processes-KNPs) (Figure 1; for further details see Boyd et al., 2025). Since these processes are essential to the developing CNS, perturbation in any of them could potentially cause developmental neurotoxic effects (Giordano and Costa, 2012; OECD, 2023). It is acknowledged that multiple molecular targets, some of which may not be well studied or understood, are involved in DNT, and it was the research and regulatory communities’ decision to come up with a pragmatic solution through the development of suitable *in vitro* assays that captures downstream converging cellular key events (KE) of multiple adverse outcome pathways (AOPs) that may be involved in the chemical exposure of the developing nervous system. Considering this complexity, it was acknowledged that a NAM-based DNT testing approach should cover a majority of these KNPs by combining multiple assays.

Over the past 20 years, considerable advancements have been made to develop assays measuring KNPs. This multiyear effort provided the foundation for an OECD project that started in 2018, which described DNT assays, provided criteria that allow evaluation of the relevance of the data to DNT, and helped determine the degree of certainty of the results to inform the use of a DNT *in vitro* battery (DNT-IVB) in regulatory hazard determinations. In April of 2023, the OECD Working Party of National Coordinators of the Test Guideline program approved document 377 that described the DNT-IVB (OECD, 2023).

The DNT-IVB comprises 17 assays that assess chemical effects using animal- and human cell culture models that measure changes in proliferation, differentiation, apoptosis, migration, neurite formation, synaptogenesis, and neural network formation (Table 1). Assays were selected based on their readiness to provide useful data for different types of regulatory scenarios (Bal-Price et al., 2018) as well as the ability of the assays to cover KNPs (Fritsche et al., 2017; OECD, 2017b). Assay developers had collectively tested a set of compounds that included DNT reference positives and negatives (Martin et al., 2022; Mundy et al., 2015) and agreed to make the data for these chemicals public available through EPA’s Comptox Chemicals Dashboard and to have completed detailed protocols in toxicity test method template (ToxTemp) forms for each of the assays (Krebs et al., 2019). The ToxTemp is an extension of OECD Guidance Document 211 (GD211) on non-guideline method documentation for regulatory purposes in chemical assessment (OECD, 2017a). The DNT-IVB performance was initially evaluated with positive and negative assay compounds, allowing mode-of-action analysis of few compounds. Assessing cell viability and/or cytotoxicity in the cell-based assays allows for the evaluation of data in terms of the selectivity of a chemical on a neurodevelopmental process (i.e., changes occurring in the absence of an overall effect on cell health or viability), providing more confidence in the relevance of *in vitro* data to perturbation of neurodevelopment. Additional information about the battery can be found in the Initial Recommendations on evaluation of data from the DNT-IVB (OECD, 2023).

Significant resources have been invested in the development of NAMs or for testing and assessing DNT, in particular by EFSA and US EPA overseen by the OECD DNT-IVB expert group. The assays included in the battery were developed at three different institutions (US EPA, The Leibniz Research Institute for Environmental Medicine (IUF), and University of Konstanz (UKN)), but many other organizations (Center for Alternatives to Animal Testing, The Division of Translational Toxicology at the National Institute of Environmental Health Sciences, Japan National Institute of Health) contributed to development, assessment and implementation of the battery.

## 3 Regulatory application of the DNT-IVB: case studies

The DNT-IVB has been applied in a growing number of regulatory case studies, primarily within Integrated Approaches to Testing and Assessment (IATA), to support chemical evaluation where traditional *in vivo* DNT data are limited or absent. These applications demonstrate how DNT-IVB data can be used in a manner suitable for regulatory decision making. These applications are consistent with conclusions from the OECD/EFSA developmental neurotoxicity workshop (Fritsche et al., 2017) and the guidance (OECD, 2023), which identified the DNT-IVB as ready for fit-for-purpose regulatory use, particularly for screening, prioritisation, and weight-of-evidence–based hazard considerations. In addition, independent scientific reviews of both individual assays and the integrated battery, have been conducted by multiple authorities and expert groups (Bal Price et al., 2018; US EPA, 2020; Hernández-Jerez, 2021; EFSA, 2022; Juberg et al., 2023). These reviews converged on the same conclusion that the DNT-IVB is sufficiently mature for fit-for-purpose aforementioned regulatory applications. This consensus has enabled regulatory bodies to draw on DNT-IVB data with an appropriate level of confidence, while acknowledging current limitations and the need for continued methodological refinement.

Early regulatory-oriented applications focused on chemical class–based assessments, including per- and polyfluoroalkyl substances (PFAS) and flame retardants (Behl et al., 2015; Carstens et al., 2023). In these case studies, DNT-IVB data were used to support prioritisation decisions, identifying substances warranting further assessment based on consistent perturbations of key neurodevelopmental processes across multiple assays (OECD, 2023). These examples established the utility of the battery as an efficient screening tool capable of supporting decisions at the early tiers of regulatory assessment. Subsequent applications extended the use of the DNT-IVB to single-chemical case studies, including deltamethrin, flufenacet, acetamiprid, glufosinate, and several organophosphate compounds (Dobreniecki et al., 2022; Hernández-Jerez, 2021; Hernández-Jerez, 2024). In these cases, DNT-IVB results were integrated with existing *in vivo* toxicological data, toxicokinetic information, and other mechanistic evidence within IATA frameworks. The DNT-IVB contributed to weight-of-evidence evaluations, helping to contextualise observed *in vivo* findings, explore biological plausibility, and support conclusions on potential developmental neurotoxicity hazards. Importantly, these applications did not treat DNT-IVB outcomes as stand-alone replacements for guideline studies, but rather as complementary evidence streams informing regulatory judgement.

To strengthen regulatory interpretation, DNT-IVB data used in case studies have generally been analysed using harmonised concentration–response modelling approaches, with raw and processed data stored in centralised databases. This has facilitated consistent interpretation across studies and increased transparency in regulatory use. Comparative evaluations of commonly applied DNT data analysis pipelines have shown a high degree of concordance, with differences mainly observed for responses near benchmark thresholds or in datasets with higher variability (Carstens et al., 2025). These findings support the robustness of conclusions drawn from DNT-IVB data in regulatory contexts, while reinforcing the importance of expert judgement for interpreting the data, in particular for borderline responses.

Addressing recommendations from regulatory reviewers, particular attention has been given to chemicals tested across the full DNT-IVB assay set, such as imidacloprid, mancozeb, methimazole, methylmercury chloride, spirodiclofen, and thiamethoxam. These substances could provide informative examples for comparing DNT-IVB outcomes with curated *in vivo* developmental neurotoxicity evidence (OECD, 2023).

Overall, the existing body of regulatory case studies demonstrates that the DNT-IVB is already being used to support decision making in clearly defined contexts, in line with the conclusions of the OECD DNT workshop (OECD, 2025a). Continued development of standardised interpretation approaches and additional case studies, is expected to further strengthen regulatory confidence and facilitate broader, more consistent use of DNT-IVB data in chemical safety assessment.

## 4 Regulatory and scientific challenges in establishing a DNT NAMs testing strategy

The nervous system comprises highly diverse, region-specific cell types whose development depends on tightly regulated spatial and temporal processes, as well as interactions with endocrine and immune systems (Conroy, 2023; Chen et al., 2013; Mangas et al., 2025b). Exposures during sensitive windows, such as prenatal and perinatal phases, can cause irreversible or delayed neurodevelopmental effects, including subtle deficits that may only emerge over time. Capturing such complexity within simplified experimental systems remains inherently challenging.

Rodent models remain widely used for DNT research and regulatory assessment, yet their ability to predict human-relevant mechanisms and phenotypes is limited (Paparella et al., 2020; van der Staay et al., 2009). While the development of the DNT-IVB represents a major advance, the battery in its current form has uncertainties when using it in isolation for DNT risk assessment (see also OECD, 2023). DNT-IVB application highlights several categories of uncertainty that are common to in vitro-based human health assessments, as well as challenges specific to neurodevelopment and the current stage of DNT-IVB regulatory maturity.

Some of these uncertainties are common with any in vitro-based human health risk assessment such as limited metabolic competence, the absence of physiological barriers such as the placenta and blood–brain barrier, and the lack of systemic interactions, including endocrine and microbiome-mediated pathways.

Additional challenges of scientific and regulatory nature are specific to the nervous system under development and the DNT-IVB, including incomplete coverage of all key neurodevelopmental processes (e.g., astrocyte maturation, myelination, and the peripheral nervous system), limited representation of complex cell–cell interactions, and the inability of *in vitro* systems to directly assess higher-order functional outcomes such as behaviour. Furthermore, no formal validation procedure has yet been completed for the DNT-IVB as an integrated battery.

Regulatory case studies applying the DNT-IVB within IATA have highlighted additional implementation challenges, including reliance on expert judgement, the absence of fully standardised workflows or tiered testing strategies, and difficulties in translating *in vitro* bioactivity into external exposure metrics. These challenges underscore the need for further standardisation of analytical pipelines, data interpretation approaches, and computational tools, particularly for exposure contextualisation and in vitro–to–in vivo extrapolation.

Several of these uncertainties are under consideration. For example, the issue of metabolism was addressed in the early 1970s when the Ames test was combined with approaches such as pre/co-treatment of compounds with liver S9 fractions (Ames et al., 1973). Similar approaches have been developed more recently for estrogen NAMs (Hopperstad et al., 2022) and could be applied to the DNT-IVB. While such approaches can increase sensitivity for detecting metabolite-mediated effects, their applicability to developmental neurotoxicity NAMs requires careful consideration. Unlike genotoxicity assays, where S9 is used primarily to maximise hazard detection in a conservative manner, neurodevelopmental processes are highly context-dependent and sensitive to experimental perturbations. Moreover, S9 systems do not recapitulate tissue-specific metabolism, placental transfer, or developmental timing, all of which are critical determinants of developmental neurotoxicity (OECD, 2023). Recent research has demonstrated that S9-based metabolisation modules can be used experimentally to explore metabolite-mediated neurotoxicity in selected NAMs (Suess et al., 2025).

A deliberate choice in the design of the DNT-IVB is that it does not address developmental neurotoxicity mediated by thyroid or other hormones, as noted in the OECD (2023) document. Similar to the issue of metabolism, potential indirect effects via endocrine disruption can be considered within WoE and IATA (Kreutz et al., 2024), tiered testing strategies, or dedicated screening programs targeting androgen, estrogen, and thyroid hormone pathways (Browne et al., 2017; Bernasconi et al., 2023; Deisenroth et al., 2025).

Currently multiple coordinated initiatives with several synergistic projects are underway to accelerate the development, standardization and regulatory use of NAMs for neurotoxicity. Within the European Union, these include large collaborative efforts such as PARC, EFSA Environmental Neurotoxicants activities, and EFSA funded Brain Health project (Tal et al., 2024; EFSA, 2025). In parallel, the OECD continues to support capacity building through guidance documents, webinars, and training materials, facilitating consistent interpretation and uptake across regulatory jurisdictions (please see also OECD webinars and training materials for further details https://www.youtube.com/playlist?list=PLJNHHjqEVIIfn6eDbh1N-tLAhxP45zBft; 2025–2026). Collectively, these activities directly address the key general and DNT-IVB-specific uncertainties identified in the OECD Initial Recommendations (OECD, 2023). While further scientific and regulatory advances are clearly needed, particularly with respect to standardisation, exposure integration, and broader biological coverage, it is recognised that refinement of the DNT-IVB and its role within DNT testing strategies will occur progressively over the coming years. These developments will proceed in parallel with broader advances in regulatory frameworks and decision-making approaches, as discussed in Section 5.

## 5 OECD activities on DNT-IVB method development and standarization

Ongoing OECD activities are focused on strengthening the scientific robustness, transferability, and standardisation of the DNT *In Vitro* Battery (DNT-IVB). These efforts aim to ensure that individual assays and the battery as a whole can be reliably implemented across laboratories, thereby supporting confidence in the generation of high-quality and reproducible data.

There is an ongoing international effort to support the transfer of all the DNT-IVB assays from the developers’ laboratories to ‘naïve laboratories’. A ‘naïve laboratory’ is a laboratory that has the technical competence and relevant chemical experience required for the test method to be transferred, but has no previous experience with this specific method or associated protocol. The initial phase of this work includes i) identification of naïve laboratories, ii) refinement and harmonization of standard operating procedures and iii) establishing a training set of compounds including both positive and negative controls. These activities have been recognized by the OECD DNT-IVB Expert Group as a critical prerequisite for advancing the current “Initial Recommendation document toward a WNT-endorsed DNT NAMs Guidance and strengthening the scientific robustness, transferability, and reproducibility of the battery.

EFSA, through a contract research organization (CRO), is contributing to this effort by providing resources and participating in demonstrations of assay transferability. In parallel, the US EPA, and the National Toxicology Program Interagency Center for the Evaluation of Alternative Toxicological Methods (NICEATM) are likewise engaged in supporting method transfer activities between US agencies and contract research organizations, thereby contributing to the broader international effort to advance DNT-IVB assay readiness. Securing sustainable global funding for further validation and standardisation of test methods, however, remains a significant challenge.

In addition to transferability, the OECD DNT-IVB Expert Group is undertaking technical activities to support the refinement and potential expansion of the existing DNT-IVB battery. Inclusion of any new assay in future iterations of the battery (e.g., DNT-IVB v2.0), they must meet the readiness criteria and description requirements outlined in the Initial Recommendations document (OECD, 2023). These criteria encompass assay maturity, standardization of protocols, availability of performance data, and clarity of biological relevance (Bal-Price et al., 2018; OECD, 2005). The Expert Group is evaluating whether candidate assays address neurodevelopmental processes not currently represented in the DNT-IVB, thereby filling identified biological or mechanistic uncertainties.

Efforts are also underway to establish an international agreed list of positive and negative DNT reference substances to support performance evaluation of individual assays and the battery as a whole and to inform potential refinements of the testing strategy, including the identification of assays that may systematically over or underpredict.

Progress in this area is constrained by i. limited availability of substances with robust human developmental neurotoxicity data; ii. relatively few chemicals have been evaluated in guideline compliant *in vivo* DNT studies, and these do not capture the full spectrum of mechanistic pathways through which chemicals may elicit DNT; iii. extracting, harmonizing, and interpreting DNT outcomes from existing *in vivo* studies is complicated by variability in study design, reporting practices, and endpoint interpretation (Mangas et al., 2025a); and; iv. only a small number of substances are currently classified as DNT under the Globally Harmonized System of Classification and Labelling of Chemicals (GHS), limiting the availability of regulatory anchored reference chemicals. Despite these limitations, substantive progress is being made through a dedicated OECD subgroup, which is focusing on the EFSA DNT Reference Chemical List proposed in the *Compendium of Information on the Use of Guideline Based Developmental Neurotoxicity Studies* (Crofton and Mundy, 2024). This work aims to support convergence toward an internationally acceptable set of reference substances that can be used to evaluate and refine the performance of the DNT-IVB. While the number of chemicals with robust human developmental neurotoxicity data remains limited and compliant *in vivo* DNT studies do not capture the full spectrum of mechanistic pathways underlying DNT, the increasing availability of curated reference data provides a valuable foundation for benchmarking battery performance. Continued refinement of reference sets, together with expanding *in vitro* datasets, is expected to progressively strengthen confidence in the evaluation of DNT-IVB performance and its applicability across regulatory contexts.

Consideration is also being given to whether data generated with established DNT reference chemicals demonstrate an improvement in the overall sensitivity, specificity, or predictive performance of the existing battery. Beyond the scope of the OECD DNT project, additional international research efforts are underway to broaden the domain of applicability of the DNT-IVB. These include the development of assays designed to capture modes of action not currently represented in the battery, such as endocrine-mediated or immune-mediated perturbations (Ramhøj et al., 2024; Lupu et al., 2020). Such developments may provide future opportunities for strategic expansion of the battery, pending evaluation against OECD readiness and performance criteria.

In addition there is a transatlantic effort to expand the chemical space and increase the number of chemical compounds tested through the battery. EFSA has supported the testing of more than two hundred compounds (EFSA, 2025). A similar number of chemicals are being evaluated by resources coming from the Division of Translational Toxicology (DTT) at the US National Institute of Environmental Health Sciences (NIEHS) (Celardo et al., 2025). Selection of chemicals for testing by the DTT was based on nominations by stakeholders representing industry, government, academia, and the public with priorities given to those known or highly suspected to have *in vivo* DNT potential. Based on the nature of existing DNT data, most of these chemicals were pesticides. However, flame retardants, industrial chemicals, food additives, and drugs were included to expand the understanding of the DNT-IVB’s applicability domain.

The US EPA has also generated data using its subset of assays included in the battery, covering approximately 250 chemicals, including around 160 of which belong to the per- and polyfluoroalkyl (PFAS) group (Carstens et al., 2023). It is worth mentioning that other organizations, such as the Danish EPA and research consortia, have supported the testing of additional compounds, albeit with more limited capacity. Regarding the curated databases, there is progress with EFSA’s compendium project, which lists publicly available studies that followed DNT and/or reproductive guidelines containing a DNT cohort (Crofton and Mundy, 2024; Mangas et al., 2025a). This compendium represents the most comprehensive available list of chemicals that have undergone *in vivo* DNT TG testing and serves as a source of information for comparing *in vivo* and *in vitro* methods, and is intended to be updated annually. It is expected that with the increasing number of chemicals exploring unsupervised machine learning (ML) Bayesian network models will allow faster analysis of the data and quantification models.

## 6 Regulatory integration of the DNT-IVB data use in chemical assessment

In parallel with methodological development, OECD activities are focused on facilitating the structured use of DNT-IVB data in chemical risk assessment. These efforts aim to support the integration of *in vitro* evidence into Integrated Approaches to Testing and Assessment (IATA), weight-of-evidence evaluations, and regulatory decision-making across different problem formulations. A new OECD project brings together work conducted within the Test Guidelines Programme and the IATA Case Studies Project to address challenges related to interpretation and regulatory application of DNT-IVB data. In addition, the new project applied physiologically based kinetic (PBK) models and developed toxicokinetic considerations specifically to evaluate chemicals that potentially alter neurodevelopment, thereby extrapolating from *in vitro* effects to concentrations that cause similar effects *in vivo* (OECD, 2025b).

The project will make use of the new IATA Framework Template, designed for cases where multiple approaches can be applied to address the same endpoint using similar information sources to address different regulatory problem formulations or for IATAs that could be adapted as a workflow for more than one chemical.

A key deliverable of this project will be adapting previous IATA Case Studies to build an IATA Framework Template specific to regulatory problem formulations related to DNT. Additional examples will be added using this new template, and supporting guidance will be developed, which are expected to increase the regulatory use of DNT-IVB data in risk assessment. The IATA Framework Template will include several testing methods (information sources in various modules) to address multiple problem formulations (i.e., regulatory decision contexts) and present a consistent way to integrate these methods depending on the problem formulation and regulatory framework.

**FIGURE 2:**
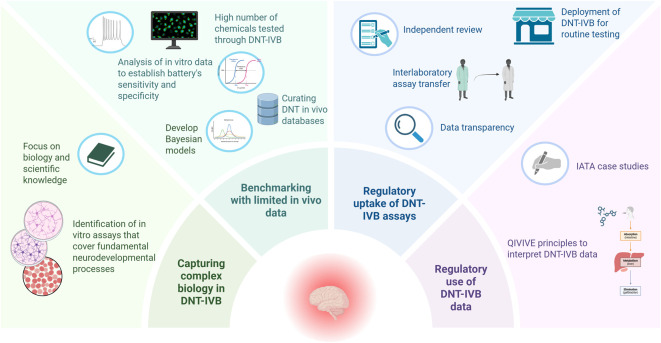
Visualisation of the main challenges in developing and using DNT-IVB in regulatory settings and collaborative efforts to address them with pragmatic approaches [DNT-IVB: DNT *in-vitro* testing battery; IATA: Integrated approaches to testing and assessment; QIVIVE: quantitative *in vitro* to *in vivo* extrapolation].

The aspiration is to increase standardization and pave the way towards the development of Defined Approaches for DNT. In contrast to the assessment process within Integrated Approaches to Testing and Assessment (IATA) that necessarily involves some degree of expert judgment, predictions generated with defined approaches are rule-based and can either be used on their own if they are deemed to be fit-for-purpose or considered together with other sources of information in the context of IATA. Since then, test guidelines that are based on defined approaches have been developed that include more than one validated method that are technologically and functionally diverse. These methods are combined to predict the same adverse effect with a fixed data interpretation procedure. The work will build on the published DNT IATA case studies and the Initial Recommendations for evaluating the DNT *In Vitro* Battery (DNT-IVB) data (OECD, 2023) and preliminary expert group work on establishing a testing workflow, guidance for quantitative *in vitro* to *in vivo* extrapolation (QIVIVE) and uncertainty analysis tables. Where possible, interpretative guidance will be developed to address considerations specific to DNT, such as QIVIVE and uncertainty analysis, to be used when an IATA is developed. The OECD workshop on “Critical innovations in pesticides safety testing and chemical risk assessment for DNT” (https://www.oecd.org/en/events/2024/10/workshop-critical-innovations-in-pesticides-safety-testing-and-chemical-risk-assessment-for-developmental-neurotoxicity.html) took place in October 2024 and brought together experts in the field to reflect on the existing DNT IATA case studies and have preliminary discussions about the DNT IATA Framework Template and QIVIVE considerations, but also share advancements in DNT testing (OECD, 2025a). Figure 2 captures challenges and solutions discussed in the present and previous sections.

## 7 Looking ahead

The active OECD projects on DNT aim to bring together critical elements and necessary guidance to support the standardization and use of NAMs to address DNT in chemical assessment.

The DNT-IVB is an example of developing a solution to a recognized gap in chemical assessment by using methods that are on the leading edge of emerging science. Rather than relying on available *in vitro* methods and repurposing these for toxicity testing, the DNT-IVB was developed based on biological knowledge and mechanistic understanding. These new assays, which formed the DNT-IVB, required addressing new challenges, for example, setting method readiness criteria to evaluate assay maturity, using stem cell technology for chemical testing, developing a battery of assays rather than stand-alone assays, and applying the IATA framework to help promote standardization that will continue for the years to come. Furthermore, it is an example of NAMs developed and integrated to address a complex endpoint and of a workflow that is sufficiently flexible to address a variety of regulatory contexts while reducing the variability associated with expert judgment. The lessons learnt from this example can be applied to external relevant projects as well as existing OECD activities in other toxicological endpoints, paving the way for a new paradigm. The DNT work also serves as an outstanding model of successful international collaboration and an ideal example of codesigning of NAMs by multiple stakeholders. In conclusion, the OECD work conducted thus far, as well as the current work underway, illustrates cooperation across OECD standardization and regulatory application activities and internationally among researchers, industry and regulators, which significantly advances regulatory application and uptake of NAMs.

## Footnotes

1 The term “defined approach” first appeared in the Guidance Document on the Reporting of Defined Approaches to be Used Within Integrated Approaches to Testing and Assessment (OECD, 2016). A defined approach consists of a fixed data interpretation procedure (DIP) (e.g., statistical, mathematical models) applied to data (e.g., *in silico* predictions, in chemico, *in vitro* data) generated using a defined set of information sources to derive a prediction.

## Author disclaimer

The contents of this publication and the opinions expressed and arguments employed herein are those of the authors and do not necessarily reflect the official views or policy of the OECD or of the governments of its member countries. The contributions of HTH are considered Works of the United States Government.
